# High Dietary Salt Intake Is Associated With Histone Methylation in Salt-Sensitive Individuals

**DOI:** 10.3389/fnut.2022.857562

**Published:** 2022-04-28

**Authors:** Yueyuan Liao, Chao Chu, Yu Yan, Dan Wang, Qiong Ma, Ke Gao, Yue Sun, Jiawen Hu, Wenling Zheng, Jianjun Mu

**Affiliations:** ^1^Department of Cardiology, First Affiliated Hospital of Medical School, Xi'an Jiaotong University, Xi'an, China; ^2^Key Laboratory of Molecular Cardiology, Xi'an Jiaotong University, Xi'an, China; ^3^Key Laboratory of Environment and Genes Related to Diseases, Xi'an Jiaotong University, Ministry of Education, Xi'an, China

**Keywords:** high salt diet, salt-sensitive hypertension, epigenetic modification, histone methylation, cohort study

## Abstract

**Background:**

High salt diet is one of the important risk factors of hypertension and cardiovascular diseases. Increasingly strong evidence supports epigenetic mechanisms' significant role in hypertension. We aimed to explore associations of epigenetics with high salt diet, salt sensitivity (SS), and SS hypertension.

**Methods:**

We conducted a dietary intervention study of chronic salt loading in 339 subjects from northern China in 2004 and divided the subjects into different salt sensitivity phenotypes. A total of 152 participants were randomly selected from the same cohort for follow-up in 2018 to explore the effect of a high-salt diet on serum monomethylation of H3K4 (H3K4me1), histone methyltransferase Set7, and lysine-specific demethylase 1 (LSD-1).

**Results:**

Among SS individuals, the blood pressure (SBP: 140.8 vs. 132.9 mmHg; MAP: 104.2 vs. 98.7 mmHg) and prevalence of hypertension (58.8 vs. 32.8%) were significantly higher in high salt (HS) diet group than in normal salt (NS) diet group, but not in the salt-resistant (SR) individuals (*P* > 0.05). Serum H3K4me1 level (287.3 vs. 179.7 pg/ml, *P* < 0.05) significantly increased in HS group of SS individuals, but not in SR individuals. We found daily salt intake in SS individuals was positively correlated with serum H3K4me1 (*r* = 0.322, *P* = 0.005) and Set7 (*r* = 0.340, *P* = 0.005) levels after adjusting for age and gender, but not with LSD-1 (*r* = −0.137, *P* = 0.251). In addition, positive correlation between the serum H3K4me1 level and Set7 level (*r* = 0.326, *P* = 0.007) was also found in SS individuals. These correlations were not evident in SR individuals.

**Conclusion:**

Our study indicates that high salt diet increases the serum H3K4me1 and Set7 levels in SS individuals.

## Introduction

Sodium is an essential nutrient, but excessive sodium intake is one of the important risk factors for hypertension (1). According to survey, dietary sodium intake in China is at a high level worldwide, and high sodium and low potassium are the major dietary feature. Recent data show that the salt intake level in China is still more than double the maximum intake recommended by the World Health Organization (2). Studies have found that individuals in the population have different blood pressure (BP) responses to salt load or salt restriction and exhibit different salt-sensitivity (SS) phenotypes (3). Studies have reported that SS individuals were not only more likely to develop hypertension than salt-resistant (SR) individuals, but also had higher rates of cardiovascular complications and mortality. High salt diet and salt sensitivity are important risk factors for hypertension and cardiovascular diseases in the population, especially in China (4).

Epigenetic mechanisms can affect gene expression and function without changing the underlying DNA sequence and can mediate crosstalk between genes and the environment (5). Increasingly strong evidence supports epigenetic mechanisms' significant role in the development of essential hypertension (6, 7). Histone methylation and demethylation play an important role in the development of environment-related diseases. Set7, as an important methyltransferase, has been shown to monomethylate H3K4 (8). Monomethylation of histone H3K4 is a signal of gene activation and plays an important role in gene transcriptional activation (9). LSD-1, induces demethylation of H3K4 or H3K9 and thereby alters gene transcription. In animal models and population studies, LSD-1 has been shown to be associated with salt sensitivity of BP (10, 11). However, the associations of histone methylation and demethylation with high salt intake, salt sensitivity, and salt-sensitive hypertension have hardly been explored.

Thus, we aimed to explore the associations of high salt intake and salt sensitive with H3K4me1, Set7, and LSD-1.

## Methods

### Study Population and Dietary Intervention

**Figure 1:**
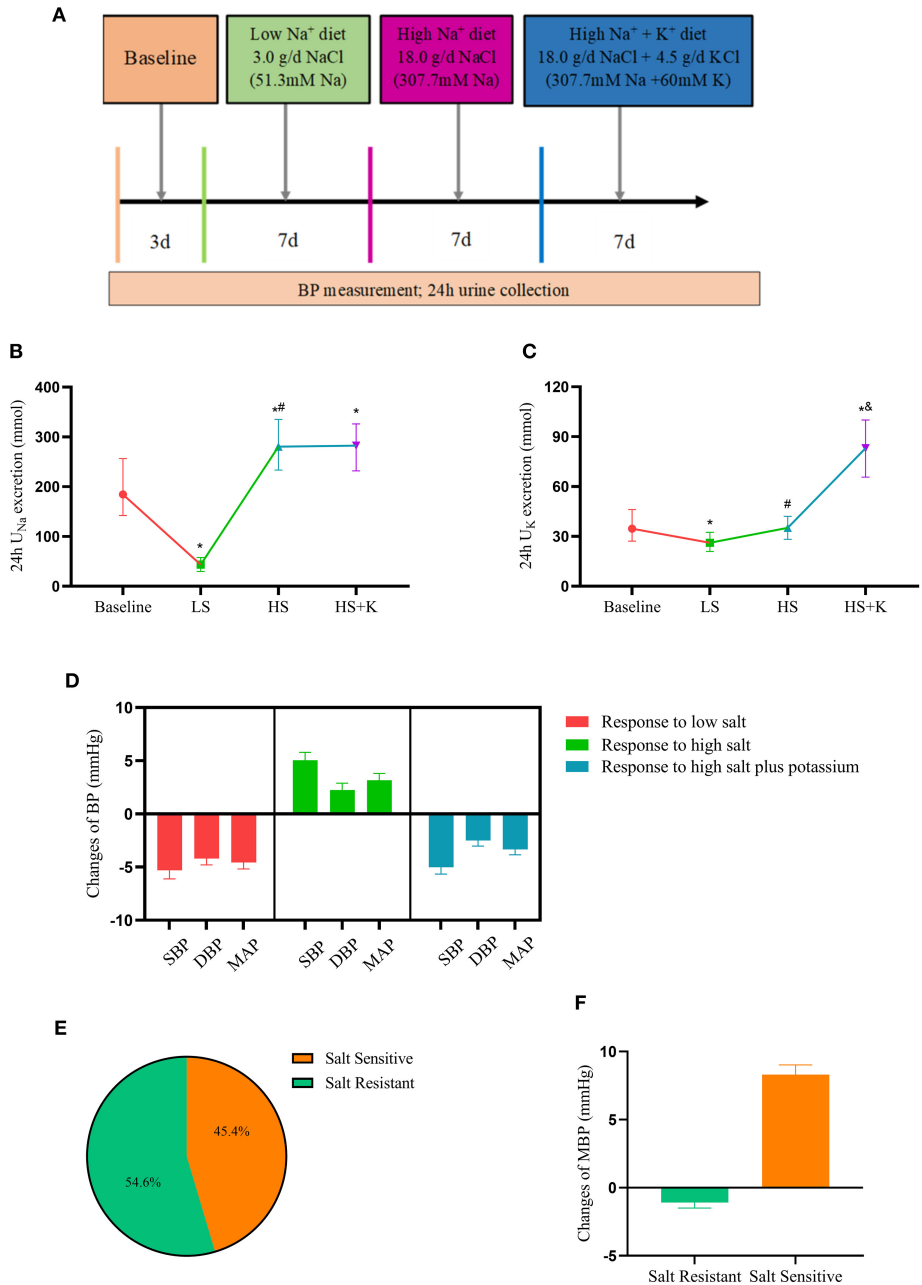
Dietary interventions in subjects-chronic salt loading trial. **(A)** The protocol of dietary intervention; **(B)** The 24 h urinary sodium excretion in participants during the dietary intervention; **(C)** The 24 h urinary potassium excretion in participants during the dietary intervention; **(D)** The responses of blood pressure to dietary intervention; **(E)** The proportion of different salt sensitive phenotypes in the participants; **(F)** The responses of MAP to high salt intervention in salt-sensitive and salt-resistant participants. BP, blood pressure; DBP, diastolic blood pressure; MAP, mean arterial pressure; SBP, systolic blood pressure; HS, high salt intervention; LS, low salt intervention; HS+K, high salt plus potassium intervention. *P < 0.05 vs. baseline; ^#^*P* < 0.05, high salt diet vs. low salt diet; ^&^*P* < 0.05, high salt plus potassium diet vs. high-salt diet.

We selected a total of 515 subjects (aged 18–60 years) for epidemiological investigation in seven villages in rural areas of Mei County, Baoji City, Shaanxi Province in 2004. Individuals who had stage 2 hypertension, secondary hypertension, severe cardiovascular disease or diabetes mellitus, liver or renal dysfunction, alcohol abuse, or pregnancy were excluded (12). Eventually, a total of 339 subjects were included in the study and a dietary intervention trial was conducted. The subjects sequentially received daily diet for 3 days, a low-salt (LS) diet (LS: 51.3 mmol/day sodium) for 7 days, a high-salt (HS) diet (307.7 mmol/day sodium) for 7 days, and a high-salt plus potassium diet (307.7 mmol/day sodium + 60 mmol/day potassium) for another 7 days (Figure 1A) (12). To ensure the dietary compliance, each participant was required to have their breakfast, lunch, and dinner in research kitchen with professional chefs under the supervision of staff.

### BP Measurement and Salt Sensitivity Definition

Subjects' BP was measured at the same time each morning by a professionally trained technician using a randomized-zero sphygmomanometer according to a standard protocol for 3 days during the baseline period and on days 5, 6, and 7 of the meal schedule. Participants were required to avoid coffee/tea, alcohol, cigarette smoking, and strenuous exercise for at least 30 min before BP measurement. Mean arterial pressure (MAP) was calculated as 1/3 systolic blood pressure (SBP) + 2/3 diastolic blood pressure (DBP). The responses of BP to dietary sodium and potassium intervention were calculated based on BP values during the dietary intervention period. Participants whose MAP increased >3 mmHg while changing from a low Na^+^ to a high Na^+^ diet were defined as SS individuals, and the other participants were defined as SR individuals (13, 14).

### Follow-Up Data Collection

A total of 152 participants were randomly selected from the same cohort for follow-up in 2018. In this study, a uniform questionnaire was used to collect information on smoking, alcohol consumption, physical activity, and relevant disease history of the subjects. At the same time, their height, weight, waist circumference and hip circumference were measured by uniformly trained staff using uniform instruments while wearing underwear and removing shoes and hats. Body mass index (BMI) = weight (kg)/height^2^ (m^2^). Sitting BP was measured in a quiet environment by trained and certified staff according to the procedures recommended by the WHO. BP was measured three times, with an interval of 2 min between each measurement, and the BP level was defined as the mean values of the three BP measurements. We performed vascular function tests on the subjects, including brachial-ankle pulse wave velocity (baPWV), ankle-brachial index (ABI), and carotid intima-media thickness (cIMT). The protocol was approved by the Ethics Committee of First Affiliated Hospital of Xi'an Jiaotong University (code: 2015–128). This study followed the principles of the Helsinki declaration and was clinically registered (NCT02734472). Written informed were obtained from each participant.

### Analysis of 24-h Urine

Urine samples were collected from subjects for 24 h to measure urine volume, and appropriate samples were retained and stored at −80°C until use. The 24 h urinary sodium, potassium and urinary microalbumin concentrations were measured in subjects. The 24 h urine sodium (24 h U_Na_, mmol) and urine potassium (24 h U_K_, mmol) were calculated by multiplying the concentration of sodium and potassium, respectively, by the 24 h total urine volume. Daily salt intake was estimated based on 24 h urine sodium excretion: daily salt intake (g) ≈ 24 h urine salt excretion (g) ≈ 24 h urine sodium excretion (mmol) ×0.0585 (g/mmol) (15). The study population were divided into four categories according to quartiles of 24 h salt intake. Individuals in the fourth quartile were defined as the high-salt diet group, while others were assigned to the normal-salt diet group for subsequent analyses.

### Serum Biochemical Analyses

Fasting venous blood samples were obtained by experienced nurses in the morning after the participants had fasted for 8–10 h. The serum isolated from the blood samples was centrifuged at a centrifugal radius of 16 cm at 3,000 r/min for 10 min at room temperature and stored at −80°C in aliquots. A Hitachi 7060 automatic biochemical analyzer was used to detect the serum biochemical parameters, including fasting glucose, serum creatinine, uric acid, total cholesterol, triglycerides, LDL cholesterol, and HDL cholesterol.

### ELISA Detection

The serum H3K4me1, Set7, and LSD-1 concentration of each subject were measured using a commercially available enzyme-linked immunosorbent assay (ELISA) kit (Xi-Tang., Shanghai, China) and is represented in pg/mL. With regard to specificity, the assay recognized and measured human serum H3K4me1, Set7, and LSD-1 without significant cross reactivity or interference with other cytokines. The sensitivity for H3K4me1, Set7, and LSD-1 were 8, 30, and 9 pg/mL, respectively. For repeatability, the intra- and inter-plate coefficients of variation were <10%.

### Statistical Analysis

Continuous variables were shown as mean ± SDs if normally distributed, otherwise, they were reported as medians (25th, 75th percentile ranges). Categorical variables were expressed as numbers and percentages of subjects. Differences between continuous variables were analyzed by the *t*-test for two-group comparisons and one-way ANOVA for three or more groups when the distribution and variance met the conditions; otherwise, the Mann–Whitney *U*-test and Kruskal–Wallis test were used. Partial correlation coefficients were calculated to evaluate the relationships of high salt intake with serum H3K4me1, Set7, and LSD-1 levels after adjusting for age and gender. The linear regression models were used to test the associations of 24 h salt intake with serum H3K4me1, Set7, and LSD-1, after adjustment for age and gender. Statistical analysis was performed using SPSS 25.0 software (SPSS Inc., Chicago, Illinois, USA). A 2-tailed *p-*value <0.05 was considered to be statistically significant.

## Results

### The 24 h Urinary Sodium and Potassium Variations During Dietary Intervention

The 24-h urine samples were collected to ensure the compliance of the subjects with the study protocol. Results showed that 24 h U_Na_ excretion significantly decreased with the change from the baseline period to the LS diet (43.5 vs. 184.4 mmol/24 h, *P* < 0.001), but increased with the change from the LS to HS diet (280.5 vs. 43.5 mmol/24 h, *P* < 0.001) (Figure 1B). Compared with the HS diet, 24 h U_Na_ excretion increased in the high salt potassium supplement period, but the difference was not statistically significant (282.3 vs. 280.5 mmol/24 h, *P* = 0.711), while 24 h U_K_ excretion was significantly increased (83.1 vs. 35.2 mmol/24 h, *P* < 0.001) (Figure 1C). These results proved the subjects' compliance with the dietary administration protocol.

### Responses of BP to Dietary Intervention

We analyzed the responses of the subjects' BP to dietary interventions. In response to LS diet intervention, SBP (−5.78 mmHg), DBP (−3.33 mmHg), and MAP (−3.63 mmHg) levels decreased; in response to HS diet intervention, SBP (+4.00 mmHg), DBP (+1.56 mmHg), and MAP (+2.37 mmHg) levels increased; in response to high-salt potassium diet intervention, SBP (−4.44 mmHg), DBP (−2.22 mmHg), and MAP (−3.04 mmHg) levels decreased again (Figure 1D). Furthermore, we divided the subjects into salt sensitive (45.4%) and salt resistant (54.6%) individuals based on their BP responses to dietary intervention (Figure 1E). The MAP reactivity was 8.31 mmHg (7.60, 9.02) in SS subjects and −1.09 mmHg (−1.49, −0.68) in SR subjects who switched from LS intervention to HS intervention (Figure 1F).

### Clinical Characteristics of Participants During Follow-Up

The demographic and clinical characteristics of the study participants by different salt-sensitive phenotypes are shown in Table 1. There were 78 SS participants and 74 SR participants. The SS participants were older than SR participants (*P* = 0.044). The heart rate (*P* = 0.025) and rate of drinking (*P* = 0.011) were higher in SS individuals than in SR individuals. There were no significant differences in gender distribution, SBP, DBP, lipid profiles, fast blood glucose, and creatinine between the SS and SR groups (all *P* > 0.05).

**Table 1:** Baseline characteristics of participants by salt sensitivity.

**Parameters**	**Total**	**Salt sensitive**	**Salt resistant**	***P*-value**
*N*, %	152	78	74	
Gender (M/F)	90 (59.2%)	44 (56.4%)	46 (62.2%)	0.471
Age (y)	54 (50, 59)	54 (50, 60)	53 (48, 57)	0.044
Height (cm)	161.6 ± 8.0	161.7 ± 8.6	161.5 ± 7.4	0.894
Weight (kg)	63.5 (56.6, 72.6)	63.2 (56.3, 72.9)	63.9 (57.2, 72.5)	0.726
BMI (kg/m^2^)	24.6 (22.7, 27.0)	24.4 (22.6, 26.8)	24.9 (23.0, 27.1)	0.532
Waist (cm)	84.0 (75.6, 91.9)	85.5 (76.8, 94.0)	82.7 (72.9, 90.2)	0.172
Hips (cm)	93.9 (89.6, 98.9)	93.0 (89.0, 99.1)	95.1 (91.0, 98.0)	0.304
SBP (mmHg)	133.5 ± 16.5	134.6 ± 14.6	132.2 ± 18.3	0.369
DBP (mmHg)	81.2 ± 10.0	82.5 ± 9.3	79.9 ± 10.7	0.108
MAP (mmHg)	98.6 ± 11.5	99.9 ± 10.4	97.3 ± 12.4	0.171
HR (bpm)	73 (67, 81)	76 (69, 82)	72 (67, 79)	0.025
Smoking (%)	56 (71.8%)	30 (38.5%)	26 (35.1%)	0.671
Drinking (%)	24 (30.8%)	18 (23.1%)	6 (8.1%)	0.011
FBG (mg/dL)	90.8 (84.7, 97.2)	90.5 (84.6, 99.0)	91.3 (85.1, 96.9)	0.867
SUA (μmol/L)	334.0 (270.5, 385.0)	347.5 (265.0, 390.1)	325.0 (272.5, 366.8)	0.224
CRE (μmol/L)	60.0 (50.0, 69.0)	59.5 (50.8, 68.0)	62.0 (48.3, 70.0)	0.958
TGs (mg/dl)	126.5 (93.7, 200.3)	146.9 (98.6, 204.2)	116.7 (87.0, 171.2)	0.087
TC (mg/dl)	172.6 (151.5, 195.4)	176.4 (157.5, 199.0)	167.4 (146.6, 192.0)	0.070
HDL-C (mg/dl)	49.1 (42.3, 59.3)	47.6 (40.6, 58.8)	49.8 (43.0, 60.5)	0.510
LDL-C (mg/dl)	88.3 (70.6, 111.8)	92.4 (71.3, 113.1)	81.0 (70.5, 107.2)	0.277
baPWV (cm/s)	1,508.0 (1,322.0, 1,777.3)	1,559.0 (1,360.0, 1,818.3)	1,473.0 (1,305.0, 1,724.0)	0.088
ABI	1.12 (1.08, 1.16)	1.12 (1.09, 1.15)	1.12 (1.07, 1.17)	0.994
CIMT (mm)	0.89 (0.83, 1.00)	0.91 (0.86, 1.00)	0.88 (0.81, 1.00)	0.081
24 h U_Na_ (mmol)	142.1 (97.2, 190.7)	137.8 (91.9, 176.1)	150.9 (103.4, 200.2)	0.262
24 h U_K_ (mmol)	29.4 (20.3, 39.0)	29.7 (20.5, 38.7)	28.6 (20.0, 39.5)	0.971
24 h Salt intake (g)	8.3 (5.7, 11.2)	8.1 (5.4, 10.3)	8.8 (6.0, 11.7)	0.262
24 h uALB (mg)	3.04 (0.00, 8.41)	4.10 (0.00, 10.40)	0.00 (0.00, 7.33)	0.165
H3K4me1(pg/mL)	200.0 (142.6, 276.8)	195.1 (142.0, 273.7)	206.8 (148.6, 281.3)	0.806
Set7 (pg/ml)	656.6 (530.1, 902.5)	693.7 (531.1, 953.7)	647.4 (527.3, 888.8)	0.643
LSD-1 (pg/mL)	340.4 (313.1, 363.9)	335.8 (311.2, 363.9)	344.1 (314.0, 365.0)	0.510

*ABI, ankle-brachial index; BMI, body mass index; baPWV, brachial-ankle pulse wave velocity; CIMT, carotid intima-media thickness; CRE, serum creatinine; DBP, diastolic blood pressure; FBG, fasting glucose; HR, heart rate; H3K4me1, monomethylation of H3K4; HDL-C, high density lipoprotein cholesterol; LSD-1, lysine-specific demethylase 1; MAP, mean arterial pressure; SBP, systolic blood pressure; SUA, serum uric acid;TGs, serum triglycerides; TC, total cholesterol; uALB, urine microalbumin; 24 h UNa, 24-hour urine sodium; 24 h UK, 24-hour urine potassium*.

### Effects of High Salt Diet on BP

The effects of high salt intake on BP level and prevalence of hypertension according to SS phenotypes are shown in Table 2. In the total population, although SBP, DBP, MAP levels, and the prevalence of hypertension in the HS diet group were slightly higher than those in the NS group, the differences were not statistically significant (*P* > 0.05). In salt sensitive individuals, SBP (140.8 vs. 132.9 mmHg, *P* < 0.05) level and MAP (104.2 vs. 98.7 mmHg, *P* < 0.05) level as well as the prevalence of hypertension (58.8 vs. 32.8%; *P* < 0.05) were significantly higher in the HS diet group than those in the NS diet group. While these differences were not found in SR individuals (*P* > 0.05). In addition, we found that among the HS diet individuals, the DBP (85.9 vs. 78.9 mmHg, *P* < 0.05) level and the prevalence of hypertension (58.8 vs. 31.3%; *P* < 0.05) were significantly higher in SS individuals than those in SR individuals. However, no differences were found among individuals with NS diets (*P* > 0.05).

**Table 2:** The effect of high salt intake on blood pressure level and prevalence of hypertension according to salt sensitivity.

	**Total**		**Salt sensitive**		**Salt resistant**	
	**NS**	**HS**	**NS**	**HS**	**NS**	**HS**
SBP (mmHg)	132.3 ± 16.1	137 ± 17.1	132.9 ± 14.5	140.8 ± 13.3^*^	131.6 ± 17.9	133.9 ± 19.5
DBP (mmHg)	81.0 ± 9.8	82.0 ± 10.7	81.6 ± 8.9	85.9 ± 10.0^#^	80.3 ± 10.9	78.9 ± 10.5
MAP (mmHg)	98.1 ± 11.2	100.3 ± 12.1	98.7 ± 10.1	104.2 ± 10.7^*^	97.4 ± 12.4	97.2 ± 12.6
Hypertension (%)	34 (29.8%)	16 (42.1%)	20 (32.8%)	11 (58.8%)^*#^	14 (26.4%)	5 (31.3%)

*DBP, diastolic blood pressure; MAP, mean arterial pressure; SBP, systolic blood pressure; NS, normal salt diet group; HS, high salt diet group*.

* *P <0.05, HS vs. NS in each group*;

# *P <0.05, Salt sensitive vs. Salt resistant in NS group and HS group*.

### Effects of High Salt Diet on Serum H3K4me1, Set7, and LSD-1 Levels

**Figure 2:**
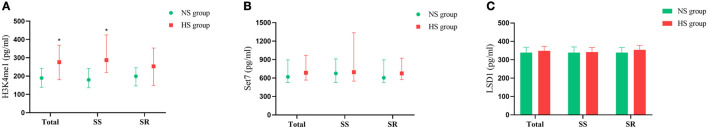
Effects of high salt diet on serum H3K4me1 **(A)**, Set7 **(B)**, and LSD-1 **(C)** in SS and SR participants. SS, salt sensitive participants; SR, salt resistant participants; NS, normal salt group; HS, high salt group. *P < 0.05, high salt group vs. normal salt group.

Figure 2 shows effects of high salt diet on serum H3K4me1, Set7, and LSD-1 levels in SS and SR individuals. In the total population, serum H3K4me1 levels (275.9 vs. 198.2 pg/ml, *P* < 0.001) were significantly increased in the HS group compared with the NS group; serum Set7 (685.5 vs. 619.7 pg/ml, *P* > 0.05) and LSD-1 (348.3 vs. 338.9 pg/ml, *P* > 0.05) levels showed an increasing trend in the HS group, but there were no statistically differences compared with the NS group. For SS individuals, serum H3K4me1 (287.3 vs. 179.7 pg/ml, *P* < 0.001) level was significantly higher in HS diet group than NS group, whereas Set7 and LSD-1 did not. For SR individuals, the high salt diet did not affect the levels of H3K4me1, Set7, and LSD-1 (*P* > 0.05).

### Correlations of 24 h Salt Intake With Serum H3K4me1, Set7, and LSD-1 Levels

**Figure 3:**
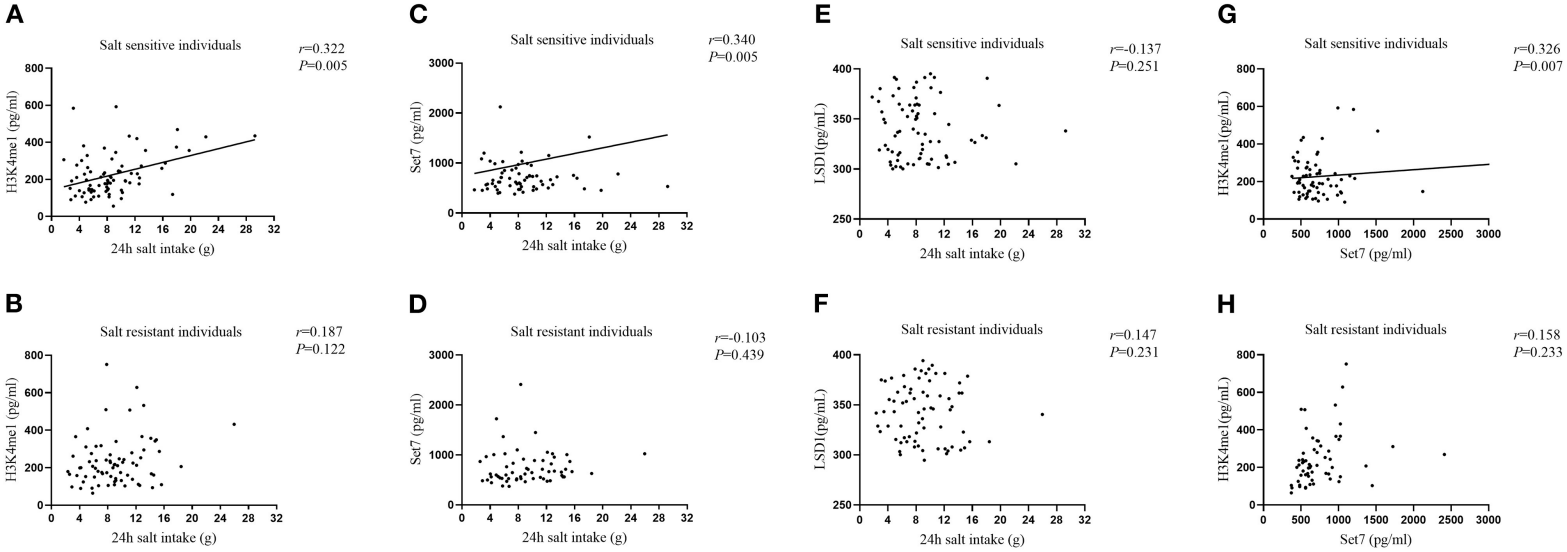
Correlations of 24 h salt intake with serum H3K4me1 in SS individuals **(A)**, H3K4me1 in SR individuals **(B)**, Set7 in SS individuals **(C)**, Set7 in SR individuals **(D)**, LSD-1 in SS individuals **(E)**, and LSD-1 in SR individuals **(F)** after adjusting age and gender. The correlation between serum H3K4me1 and Set7 in SS individuals **(G)** and in SR individuals **(H)** after adjusting for age and gender.

We examined correlations of 24 h salt intake with serum H3K4me1, Set7, and LSD-1 levels by partial correlation analysis. As shown in Figure 3, we found that 24 h salt intake in SS individuals was positively correlated with serum H3K4me1 (*r* = 0.322, *P* = 0.005) and Set7 (*r* = 0.340, *P* = 0.005) levels after adjusting for age and gender, but not with LSD-1 (*r* = −0.137, *P* = 0.251). In addition, positive correlation (*r* = 0.326, *P* = 0.007) between serum H3K4me1 level and Set7 level was also found in SS individuals. These correlations were not evident in SR individuals (*P* > 0.05).

Furthermore, we analyzed associations of 24 h salt intake with serum H3K4me1, Set7, and LSD-1 levels in individuals with different salt sensitive phenotypes using linear regression models (Table 3). The 24 h salt intake in SS individuals was significantly associated with serum H3K4me1 (β = 0.348, *P* = 0.005) and Set7 (β = 0.366, *P* = 0.005) levels after adjusting for age and gender, but not with LSD-1. These associations were not found in SR individuals (*P* > 0.05).

**Table 3:** Associations of 24 h salt intake with serum H3K4me1, Set7, and LSD1 according to salt sensitivity.

	***B*-value**	***β-*value**	***t*-value**	**95% CI**	***P*-value**
**Salt sensitive**					
H3K4me1 (pg/mL)	9.158	0.348	2.868	2.79–15.53	0.005
Set7 (pg/mL)	93.816	0.366	2.913	29.50–158.13	0.005
LSD1 (pg/mL)	−1.025	−0.148	−1.159	−2.79–0.74	0.251
**Salt resistant**					
H3K4me1 (pg/mL)	5.558	0.184	1.566	−1.52–12.64	0.122
Set7 (pg/mL)	−13.525	−0.102	−0.779	−48.28–21.23	0.439
LSD1 (pg/mL)	0.974	0.144	1.209	−0.64–2.58	0.231

*The linear regression models were used to test the associations of 24 h salt intake with serum H3K4me1, Set7, and LSD1, after adjustment for age, sex*.

## Discussion

In the present study, we analyzed for the first time the effect of a high salt diet on serum histone methylation as well as methylation-related enzymes in SS individuals using a population cohort in Mei County, Shaanxi Province. We found that a high salt diet increased BP levels and the prevalence of hypertension in SS individuals but not in SR individuals. More importantly, we found significantly positive correlations of 24 h salt intake with serum H3K4me1 and Set7 levels except for LSD-1 in SS individuals, whereas these associations were not found in SR individuals.

Studies have found that individuals in the population have different BP responses to salt load or salt restriction and exhibit different salt sensitivity phenotypes. We conducted a dietary intervention trial with subjects in the order of baseline period, low salt intervention, high salt intervention and high salt plus potassium intervention. The study found that subjects had higher urinary sodium excretion and lower urinary potassium excretion at baseline, which reflecting the dietary characteristics of high sodium and low potassium in this region. This is also in line with the reported dietary habits of rural residents in northern China (16). In addition, we found that the BP level of the subjects in the high salt intervention period was not significantly different from those at baseline. However, the subjects' BP response to dietary sodium-potassium intervention was normal, which again proved the high salt characteristics of the subjects' daily diet. These findings are similar to the result of a meta-analysis of 70 studies in 2019. The study reported that the average sodium and potassium excretion of individuals over 16 years of age in China were 189.07 mmol/24 h and 36.35 mmol/24 h, respectively, and was significantly higher in the north than in the south (2). Therefore, it is of great significance to conduct an epidemiological study of high salt diet and explore its impact on hypertension and related diseases, especially for residents in northern China.

Salt sensitivity is an intermediate phenotype of hypertension that results from the interaction of genetic and environmental factors (17). Studies have suggested that SS individuals were more likely to develop hypertension and ultimately cardiovascular diseases than SR individuals (4, 18). Our study found that the BP level and prevalence of hypertension were significantly higher in the high salt diet group than in the normal salt diet group among SS individuals, but not in SR individuals. In addition, SS individuals had higher BP levels and higher prevalence of hypertension than SR individuals in the high salt diet, but no difference in the normal salt diet. These results suggest that SS individuals have a higher risk of hypertension in the presence of a high salt diet. Therefore, in SS individuals, reducing sodium intake is of great significance for reducing the risk of hypertension.

Histones are important proteins responsible for maintaining the structure of chromatin and play a role in the dynamic and long-term regulation of genes. The N-terminal tail of histone 3, one of the five histones found in eukaryotic nuclei, is subject to methylation or acetylation of lysine and arginine residues as well as phosphorylation of serine and threonine residues (19). Monomethylation of histone H3K4 is a signal of gene activation and plays an important role in environmentally induced diseases. Our study found that 24 h salt intake in SS individuals was positively correlated with serum H3K4me1 and Set7 levels. In addition, positive correlation between the serum H3K4me1 level and Set7 level was also found in SS individuals. These correlations were not evident in SR individuals. Various findings suggest that epigenetic mechanisms have an important role in obesity-induced hypertension. Obesity-induced hypertension is associated with increased levels of histone deacetylase 1 and acetylated histone H3 in mice fed a high-fat diet (20). In addition, previous study had found the epigenetic modulation of WNK4 transcription in the development of salt sensitive hypertension (21). Histone deacetylation has also been found to play an important role in angiotensin II-induced hypertension (22). The result of this study suggest that a high-salt diet increases the expression of the lysine methyltransferase Set7 and increases the monomethylation of lysine at position 4 of histone H3 in salt sensitive individuals. And these changes may lead to a series of related diseases. Then, exploring histone methylation may be important for the prevention and treatment of salt sensitive hypertension.

LSD-1 is an epigenetic regulator of gene transcription, and it has been demonstrated to be associated with salt sensitivity of BP. Luminita et al. found that LSD-1 deficiency is associated with increased BP and vascular reactivity in mice during liberal salt intake, suggesting that salt intake has an epigenetic effect (10). Similar to LSD-1^+/−^ mice, African-American minor allele carriers of two LSD-1 SNPs displayed greater change in SBP in response to change from low to liberal salt diet (11). Differently, in our study, there was no association between high salt diet and serum LSD-1, neither in SS individuals or SR individuals. There may be two reasons for this, one may be ethnic differences, and the other may be that our study did not explore SNPs but only analyzed protein levels.

Epigenetic modifications alter gene function without altering gene sequence, with heritable and sustainable properties. This property may also explain the results of some studies. For example, the well-known THOP study has demonstrated that sodium reduction can lower BP (23), and also reduce long-term risk of cardiovascular events. People with prehypertension assigned to a sodium reduction intervention experienced a 25–30% lower risk of cardiovascular outcomes in the 10–15 years after the trial. Furthermore, some scholars have studied and explored this phenomenon (24). Oguchi et al. (25) showed that transient high salt intake during early phases in the development of hypertension induced sustained elevation of BP in hypertensive model rats and first named this phenomenon salt memory. More and more researches show that epigenetics play an important role in the pathogenesis of metabolic memory (26, 27). In this study, we found an association between high salt intake and histone methylation in salt sensitive individuals. Whether epigenetic changes are also involved in the formation and regulation of salt memory effect deserves further exploration.

The greatest strength of this study is the first exploration of the effects of a high-salt diet on serum histone methylation and related enzymes in a population with different salt sensitive phenotypes. This finding is important because it provides evidence for dietary salt affecting epigenetic modification. There are, of course, some limitations to this study. Firstly, the recruited population in this study was restricted to northern Chinese individuals and relatively small. Therefore, we could not generalize the results of this study to other races. Multiethnic clinical trials were required to determine whether our results could be generalized to populations with multiple backgrounds. Secondly, we focused on changes in histone methylation and related enzymes at the protein expression level under a high-salt diet. Next, we can deeply explore the changes in SNPs of these proteins. Finally, further studies are warranted to elucidate the genes modified by methylation and the exact mechanisms.

## Conclusion

In conclusion, the present study indicated that a high-salt diet was associated with serum H3K4me1 and Set7 levels in salt sensitive individuals but not in salt resistant individuals. This finding provides evidence for high salt diet affecting epigenetic modification and may have potential clinical and public health implications.

## Data Availability Statement

The raw data supporting the conclusions of this article will be made available by the authors, without undue reservation.

## Ethics Statement

The protocol was approved by the Ethics Committee of First Affiliated Hospital of Xi'an Jiaotong University (code: 2015–128). This study followed the principles of the Helsinki declaration and was clinically registered (NCT02734472). The patients/participants provided their written informed consent to participate in this study.

## Author Contributions

JM conceived, designed the study, and secured funding. YL analyzed the data. YL and CC wrote the first draft of the manuscript. QM, KG, YS, JH, and WZ supervised the data collection. YY and DW provided technical direction and writing assistance in the preparation of this manuscript. All authors critically revised the manuscript and approved the final version to be published.

## Funding

This work was supported by the National Natural Science Foundation of China No. 81870319 (JM) and No. 81700368 (CC), Grant 2017YFC1307604 from the Major Chronic Non-communicable Disease Prevention and Control Research Key Project of the Ministry of Science and Technology of the People's Republic of China, the Clinical Research Award of the First Affiliated Hospital of Xi'an Jiaoton University (XJTU1AF-CRF-2019-004), and Grant 2017ZDXM-SF-107 from the Key Research Project of Shaanxi Province. National Key R&D Program of China (2016YFC1300104).

## Conflict of Interest

The authors declare that the research was conducted in the absence of any commercial or financial relationships that could be construed as a potential conflict of interest.

## Publisher's Note

All claims expressed in this article are solely those of the authors and do not necessarily represent those of their affiliated organizations, or those of the publisher, the editors and the reviewers. Any product that may be evaluated in this article, or claim that may be made by its manufacturer, is not guaranteed or endorsed by the publisher.
